# Routine Use of Neck Drains Following Thyroid Operations to Prevent Complications Is No Longer Advisable

**DOI:** 10.7759/cureus.54388

**Published:** 2024-02-18

**Authors:** Gonçalo Guidi, Carlos Santos, João Pinto-de-Sousa

**Affiliations:** 1 General Surgery, Centro Hospitalar de Trás-Os-Montes e Alto Douro, Vila Real, PRT; 2 Surgery, Clinical Academic Centre Trás-Os-Montes e Alto Douro, Vila Real, PRT

**Keywords:** cervical drainage, general surgery complication, cervical hematoma, surgical drains, thyroid surgery complications

## Abstract

Background: The use of cervical drains to prevent cervical hematoma or seroma after thyroidectomy remains a controversial issue.

Objective: Identify clinical and surgical risk factors for hematoma or seroma and evaluate the usefulness of routine use of drains following thyroid surgery.

Material and methods: The authors conducted a retrospective multicentric study related to consecutive patients submitted to thyroid surgery in seven Portuguese hospitals between January 2018 and December 2020 (n=945). The data collected included the following parameters: age and gender of the patients, anticoagulation or anti-aggregating therapy, histological diagnoses, type of surgery, the presence or absence of postoperative drains, thyroid weight, length of hospital stay, postoperative complications, and reinterventions. In this study, surgical complications evaluated were limited to the presence of hematoma or seroma.

A total of 945 patients who underwent thyroid surgery were included in the study. Twenty-seven patients (2.9%, n=27) experienced complications classified as hematomas or seromas. In the series, significant differences were observed between the two groups according to hypocoagulation or anti-aggregation status (OR=3.62; 95% CI 1.14-11.4) (p=0.001) and the nature of histological diagnosis (toxic vs. non-toxic benign disease) (OR=6.59; 95% CI 1.83-23.7). Hypocoagulation or anti-aggregation status were independently associated with a higher risk of complications. The presence of drains was associated with longer hospitalization periods (p<0.001) and not a decreased need for reintervention.

Conclusion: Cervical hematoma or seroma are rare complications associated with both hypocoagulation and anti-aggregation therapy and with the presence of benign toxic pathology. The use of drains does not decrease the need for reintervention and is even associated with a longer length of hospital stay; therefore, their routine use should not be advised.

## Introduction

Operations on the thyroid are the most common surgical procedures performed in the neck [1]. However, the routine use of drains following thyroid surgery is still controversial [2-5]. Supporters of routine drainage argue that drains will reduce post-operative collections and reduce the likelihood of hematomas or seromas that may cause compression of the airway or become infected [6,7].

On the other hand, authors who do not use drains argue that drains often become blocked, which limits their usefulness [2], which could increase the risk of infection, the length of hospital stay, treatment costs, and discomfort for the patient. Moreover, the routine use of drains should not be a substitute for meticulous surgical technique with careful hemostasis [1,8-10].

The objective of the present study is to identify clinical and surgical risk factors for hematoma or seroma and to evaluate the usefulness of routine use of drains following thyroid surgery.

## Materials and methods

A retrospective multicentric study aimed to identify clinical and surgical risk factors for hematoma or seroma and to evaluate the usefulness of routine use of cervical drains was undertaken.

Inclusion and exclusion criteria

Consecutive patients submitted to thyroid surgery in seven Portuguese hospitals (Centro Hospitalar De Trás-Os-Montes E Alto Douro, EPE; Hospital da Horta, EPE; Hospital Distrital de Santarém, EPE; Unidade Local de Saúde do Litoral Alentejano, EPE; Hospital do Espírito Santo de Évora, EPE; Centro Hospitalar de Entre Douro e Vouga, EPE; Centro Hospitalar do Oeste, EPE - Unidade de Caldas da Rainha) between January of 2018 and December of 2020 were included.

Patients whose required information was missing were excluded from the study. No minimal age was used to exclude patients. Before data collection, all hospitals included in this study were granted approval from their ethical boards. A total of 945 patients undergoing thyroid surgery (hemithyroidectomy, total thyroidectomy, and totalization of thyroidectomy), with or without lymph node dissection, between the periods afore indicated were included in the study. Clinical and outcome data were collected, including the age and gender of the patients, anticoagulation or anti-aggregating therapy, histologic diagnoses, type of surgery, post-operative drains, thyroid weight, length of hospital stay, postoperative complications, and the need for reintervention.

The complications evaluated in this study were the presence of hematoma or seroma. The diagnoses considered were documented on the final pathology report of the surgical specimen, and accordingly, cases were divided into toxic benign pathology (toxic nodule, toxic multinodular goiter, and Graves' disease), non-toxic benign pathology, and malignant pathology. The cases were cataloged in two groups according to the presence of complications, which were compared according to the parameters evaluated.

Data analysis

The data were analyzed with the STATA 15.1 statistical package computer program (StataCorp LLC, Texas, USA). Continuous variables are expressed as the median value. Categorial variables are presented as percentages. Continuous variables were compared by the Mann-Whitney-U test and categorical variables by the Fisher’s exact test. A multivariable logistic regression model was created to identify factors that are associated with the occurrence of complications in patients undergoing thyroid surgery. The results were expressed as an odds ratio (OR) value and its 95% confidence interval (CI). The significance level was set at 0.05.

## Results

A total of 945 patients undergoing thyroid surgery in the seven hospitals that conducted the study were included: 303 patients in 2018, 298 in 2019, and 344 in 2020.

Cases were cataloged in two groups according to the presence of complications related to thyroid surgery. The distribution of cases between the two groups considered according to the several parameters evaluated in the study is summarized in Table 1. Significant differences between the groups were observed according to the age of the patients (p=0.002), the status of antithrombotic therapy (p=0.001), and the need for reintervention (p<0.001). The age of the patients with complications (68.0 years old) was higher compared to that of those without complications (57.0 years old) (p=0.002). The percentage of patients who were under antithrombotic therapy was higher in cases with complications (25.9%, n=7) compared to that of cases without complications (10.1%, n=93) (p=0.001). The percentage of patients submitted to reintervention was higher in the complications group (33.3%, n=9) compared to that observed in cases without complications (1.6%, n=15) (p<0.001).

**Table 1:** Sociodemographic, clinical, and surgical characteristics of the patients included in the study, comparing the two groups of patients according to the presence of complications

	n (%) or median (P25-P75)	No complications	Complications	p-value
	945	918 (97.1)	27 (2.9)	
Sociodemographic characteristics				
Age (years)	57.0 (46.0–68.0)	57.0 (46.0–68.0)	68.0 (57.0–76.0)	0.002
Gender				0.602
Female	786 (83.2)	762 (83.0)	24 (88.9)	
Masculine	159 (16.8)	156 (17.0)	3 (11.1)	
Clinical and surgical characteristics				
Antithrombotic therapy				0.001
No	845 (89.4)	825 (89.9)	20 (74.1)	
Anti-aggregation	75 (7.9)	73 (7.9)	2 (7.4)	
Hypocoagulation	25 (2.7)	20 (2.2)	5 (18.5)	
Diagnostic				0.083
Non-toxic benign pathology	650 (68.8)	633 (68.9)	17 (63.0)	
Toxic benign pathology	91 (9.6)	85 (9.3)	6 (22.2)	
Malignant pathology	204 (21.6)	200 (21.8)	4 (14.8)	
Surgery				0.822
Thyroidectomy	509 (53.9)	493 (53.7)	16 (59.3)	
Totalization of thyroidectomy	32 (3.4)	32 (3.5)	0 (0.0)	
Hemithyroidectomy	404 (42.7)	393 (42.8)	11 (40.7)	
Thyroid weight (g)	29.0 (16.0–53.0)	29.0 (16.0–52.0)	38.5 (23.6–88.0)	0.104
Hospital admission				0.228
Hospitalized	645 (68.3)	624 (68.0)	21 (77.8)	
Ambulatory	90 (9.5)	90 (9.8)	0 (0.0)	
Overnight stay	210 (22.2)	204 (22.2)	6 (22.2)	
Reintervention				<0.001
No	921 (97.5)	903 (98.4)	18 (66.7)	
Yes	24 (2.5)	15 (1.6)	9 (33.3)	
Lymph node dissection				0.570
No	926 (97.99)	899 (95.13)	27 (2.86)	
Yes	19 (2.01)	19 (2.01)	0 (0.0)	

No significant differences were observed in the comparison between the two groups according to the other parameters evaluated. Although there was no statistically significant difference, the relative frequency of cases with complications whose diagnosis was benign toxic pathology was higher when compared to the proportion of patients with the same diagnosis and without complications (22.2%, n=6 vs. 9.3%, n=85) (p=0.083).

The results of the multivariate logistic regression to identify independent factors associated with the occurrence of complications are summarized in Table 2. As identified in Table 2, antithrombotic therapy had a significant association (OR=3.62; 95% CI: 1.14-11.4). The presence of toxic, benign pathology documented in the surgical specimen was also identified as an independent factor in the occurrence of complications.

**Table 2:** Logistic regression analysis to identify the parameters associated complications in patients undergoing thyroid surgery

	Odds ratio (IC 95%)
Age (years)	1.04 (1.00–1.09)
Thyroid weight (g)	1.00 (0.99–1.01)
Antithrombotic therapy	
No	1
Hypocoagulation or anti-aggregation	3.62 (1.14–11.4)
Pathology	
Non-toxic benign pathology	1
Toxic benign pathology	6.59 (1.83–23.7)
Malignant pathology	2.24 (0.60–8.32)

The frequency of cervical drainage in several hospitals ranged between 6.25% and 100%. In the global series, cases were also cataloged in two groups according to the use of cervical drains: with or without drains (35.3%, n=334 vs. 64.7%, n=611, respectively). Table 3 summarizes the distribution of cases between the groups considered based on the evaluated parameters. Significant differences were observed according to histologic diagnosis (p<0.001), the type of surgery performed (p<0.001), thyroid weight (p<0.001), the regimen of admission (p<0.001), and the need for cervical lymph node dissection (p=0.002). A trend of significance was observed in the comparison between the groups according to the need for reintervention (p=0.053). No differences were observed in the distribution of cases according to the other evaluated parameters. The percentage of cases with the use of drains was higher in non-toxic benign pathology (75.1%, n=251) compared to that observed in cases without drains (65.3%, n=399) (p<0.001). In the series, the percentage of cases with drains in total thyroidectomy was higher than that of cases without drains (64.1%, n=214 vs. 48.3%, n = 295), respectively. In cases of hemithyroidectomy, the reverse was observed (34.4%, n=115 vs. 47.3%, n=289, respectively). In cases where cervical drains were used, the median weight of thyroid specimens was higher (37 g vs. 26 g, respectively). There was a significant difference in the length of hospital stays: 56.6% (n=346) of patients without a drain were hospitalized for 0-1 days, while 94.3% (n=315) of patients with a drain were hospitalized for two days or more. The percentage of cases in which a drain was used in cervical lymphadenectomy (3.9%, n=13) was higher than that in cases without lymphadenectomy (1.0%, n=6). Regarding the need for reintervention, a trend toward a significant difference was observed between patients who had a drain placed and those who did not (1.2%, n=4 vs. 3.3%, n=20; p=0.054).

**Table 3:** Summary of distribution of sociodemographic, clinical, and surgical characteristics between the two groups of patients according to the presence of cervical drains.

	No drain	Drain	p-value
	611 (64.7)	334 (35.3)	
Sociodemographic characteristics			
Age (years), median (P25-P75)	57 (45–68)	58 (47–68)	0.114
Gender			0.344^‡^
Female	503 (82.3)	283 (84.7)	
Masculine	108 (17.7)	51 (15.3)	
Clinical and surgical characteristics			
Antithrombotic therapy			0.588^‡^
No	551 (90.2)	294 (88.0)	
Anti-aggregation	45 (7.4)	30 (9.0)	
Hypocoagulation	15 (2.4)	10 (3.0)	
Diagnostic			<0.001^‡^
Non-toxic benign pathology	399 (65.3)	251 (75.1)	
Toxic benign pathology	75 (12.3)	16 (4.8)	
Malignant pathology	137 (22.4)	67 (20.1)	
Surgery			<0.001
Thyroidectomy	295 (48.3)	214 (64.1)	
Totalization of thyroidectomy	27 (4.4)	5 (1.5)	
Hemithyroidectomy	289 (47.3)	115 (34.4)	
Complications			0.316^‡^
No	596 (97.5)	322 (96.4)	
Yes	15 (4.5)	12 (3.6)	
Thyroid weight (g), median (P25-P75)	26 (15–46)	7 (21–69)	<0.001
Days of admission			
0 to 1 day	346 (56.6)	19 (5.7)	
2 or more days	265 (43.4)	315 (94.3)	
Reintervention			0.054
No	591 (96.7)	330 (98.8)	
Yes	20 (3.3)	4 (1.2)	
Lymph node dissection			0.002^‡^
No	605 (99.0)	321 (96.1)	
Yes	6 (1.0)	13 (3.9)	

## Discussion

Patients with thyroid surgery may experience complications ranging from minor to life-threatening, namely hematoma or seroma leading to neck compression and respiratory failure. The use of cervical drains has been a matter of debate in the literature, and some controversy remains. Some authors advise the routine use of cervical drains to prevent the occurrence of such complications [11], but others claim that drains are not needed or maybe more deleterious for the patient [9,12].

This study aimed to identify the utility of cervical drains in the population submitted to thyroid surgery in seven Portuguese hospitals. The study is multicentric, which may bias the interpretation of the results due to some inevitable heterogeneity of the procedures, surgical team experience, indications or options for the use of drains (ranges between 6.25% and 100%), and the identification of complications. 

First, the study claims a low percentage of complications theoretically preventable by cervical drains, which were, by definition, hematomas or seromas. Although the frequency of complications is low in the study (2.9%, n=27), it is slightly higher than the data described in other literature reports (incidence of 0.1% to 1.1%) [13].

In the series, complications were mainly associated with the age of patients and the use of antithrombotic therapies. Indeed, in this series, despite the use of SPA (Portuguese Society of Anesthesiology) guidelines for the management of these patients, antithrombotic therapy was an independent factor in the occurrence of hematomas or seromas following thyroid surgery. These results are in agreement with the literature, where patients on anticoagulation are at a greater risk for bleeding [14].

The results of this study point to a higher level of complications in patients with benign toxic pathology as opposed to those observed in cases of non-toxic benign pathology and even malignancies. Indeed, enhanced vascularization verified in such cases can explain the complications observed and the increased use of drains in these cases. The literature in these cases is ambiguous. While some advocate that hyperthyroidism is a risk factor for hematoma [14-16], others failed to identify the same relationship [17,18].

The presence of a cervical drain did not seem to be associated with the occurrence of complications or with the need for reintervention. As observed, no significant association between the presence of cervical drains and the occurrence of complications was observed, which agrees with other reports in the literature [5,19-23]. The results of this study also showed that of the patients who had complications, 12 (44.4%) had had a drain placed. Indeed, no statistically significant differences were observed between patients who had a drain placed and those who did not (1.2%, n=4 vs. 3.3%, n=20; p=0.054) regarding the need for reintervention. These results agree with those of others [24].

The study identified a higher length of hospital stay in those who had drains, with 56.6% (n=346) of patients without a drain being hospitalized for 0 to 1 day, while 94.3% (n=315) of patients with a drain were hospitalized for two days or more (p<0.001). These results agree with those observed in other studies [1,4] and can be explained by the more liberal use of drains in more thyroid operations performed in the admission regime, partially explained by the anticipation of more complications from the surgical procedures. As the use of drains did not decrease the need for reintervention and was even associated with a longer hospital stay, the routine use of cervical drainage after thyroidectomy should not be advised. This conclusion, based on the present data, is consistent with prospective studies reported in the literature [4,5,12,19-23,25,26]. The results of this study identified a higher frequency of thyroid surgery, cervical lymph node dissection, and the option of cervical drainage. The results observed did not influence the need for reoperations compared to cases without drain placement [27]. Therefore, based on this and other studies, we should not recommend routine use of cervical drains [4,27]. This study includes a significant number of patients who underwent surgery but has several limitations. It is a retrospective and multicentric study, which may be associated with a heterogeneity of procedures and diverse criteria for the use of cervical drains. Due to the low number of complications, it is possible that some associations were not found.

## Conclusions

Summing up, the percentage of patients who develop complications in this type of surgery is low, which should be considered a limitation in the interpretation of the results. However, it is recommended to limit the routine use of drains to patients under antithrombotic therapy or those with a benign toxic pathology. As the use of drains did not decrease the need for reintervention and was even associated with a longer hospital stay, the routine use of cervical drainage after thyroidectomy, from our point of view, should not be advised.

We hope this article can be the basis for a multicentric prospective study centered on patients with complications, with unified surgical protocols to evaluate this matter.
